# Arrhythmogenic cardiomyopathy with left ventricular involvement versus ischemic heart disease: lessons learned from the family study and the reviewed autopsy of a young male

**DOI:** 10.1080/20961790.2019.1616247

**Published:** 2019-08-19

**Authors:** Pilar Molina, Jorge Sanz-Sánchez, Manuel Fenollosa, Marina Martínez-Matilla, Juan Giner, Esther Zorio

**Affiliations:** a Servicio de Patología, Instituto de Medicina Legal y Ciencias Forenses de Valencia, Valencia, España;; b Unidad de Valoración del Riesgo de Muerte Súbita Familiar and Unidad de Cardiopatías Familiares, Muerte Súbita y Mecanismos de Enfermedad (CaFaMuSMe), Instituto de Investigación Sanitaria La Fe, Valencia, España;; c Servicio de Cardiología, Hospital Universitario y Politécnico La Fe, Valencia, España;; d Unidad Genómica, Instituto de Investigación Sanitaria La Fe, Valencia, España

**Keywords:** Forensic sciences, forensic pathology, ischemic heart disease, arrhythmogenic cardiomyopathy, family study

## Abstract

Ischemic heart disease (IHD) is the leading cause of sudden cardiac death (SCD) and often non-thrombosed severe coronary stenoses with or without myocardial scars are detected. Left dominant arrhythmogenic cardiomyopathy (LDAC) is a life-threating rare disease which has been more thoroughly studied in the last 10 years. The macroscopic study of an SCD victim was conducted and re-evaluated 9 years later. The cardiological work-up in his first-degree relatives initially comprised an electrocardiogram (ECG) and an echocardiogram. When they were re-evaluted 9 years later, a cardiac magnetic resonance, an ECG-monitoring, an exercise testing and a genetic study were performed and the pedigree was extended accordingly. In 2008, an IHD was suspected in the sports-triggered SCD of a 37-year-old man upon the postmortem (75% stenosis of the left main and circumflex coronary arteries; the subepicardial left ventricular fibrofatty infiltration with mild myocardial degeneration was assumed to be a past myocardial infarction). No cardiomyopathy was identified in any of the two proband’s sisters. Nine years thereafter, distant relatives were diagnosed with LDAC due to a pathogenic desmoplakin mutation. The reanalysis of the two sisters showed ventricular arrhythmias in one of them without structural heart involvement and the reviewed postmortem of the proband was reclassified as LDAC based on the fibrofatty infiltration; both were mutation carriers. The completion of the family study on 19 family members yielded one SCD due to LDAC (the proband), three living patients diagnosed with LDAC (two with a defibrillator), one mutation carrier without structural ventricular involvement, and 14 healthy relatives (who were discharged) with a very good co-segregation of the mutation. Although rare, LDAC exists and sometimes its differential diagnosis with IHD has to be faced. Modifying previous postmortem misdiagnoses can help family screening to further prevent SCDs.

## Introduction

Sudden cardiac death (SCD) is the leading cause of unexpected natural sudden deaths in developed countries [1, 2] accounting worldwide for 4.5 million cases each year [3]. Among cardiac causes, ischemic heart disease (IHD) is the most frequent cardiac condition causing SCD [1, 2]. The Association of European Cardiovascular Pathology (AECVP), aware about the potential value of a thorough postmortem investigation to determine the precise cause of the death, has released updated guidelines to be followed in this scenario [4, 5]. In them, the cause of death can be established as IHD for certain when an acute myocardial infarction is observed at the macroscopic and histological heart examination and with a high probability when only chronic IHD with ischemic scar and/or an atherosclerotic plaque causing at least 75% of lumen reduction are observed [5].

Arrhythmogenic cardiomyopathy (AC) is an inherited rare disease characterized by a progressive myocardial fibrofatty replacement [6]. A growing list of genes is now associated with this condition (Table 1). Mutations in the five desmosomal genes (*PKP2*, *DSG2*, *DSP*, *DSC2* and *JUP*) were identified in affected individuals [7–9]. As in other cardiomyopathies, the penetrance can be incomplete and its clinical expressivity variable among mutation carriers [6]. Its clinical diagnosis is not straightforward, instead a set of major and (or) minor criteria (comprising electrical, arrhythmic, structural, histological and familial/genetic items) need to be met to confirm the diagnosis [10]. Ventricular arrhythmias may cause SCD, sometimes as the first sign of alarm in a given family. Thus, the autopsy of a deceased proband is of paramount importance since it can unmask the underlying genetic disease in a given family and more affected relatives may benefit of an early diagnosis and treatment. According to the updated AECVP guidelines, the identification of the histological features of the disease (fibrofatty myocardial substitution with replacement fibrosis and myocardial degeneration) in an SCD postmortem would yield AC as the cause of the SCD with a high probability [5].

**Table 1.** Genetic background of arrhythmogenic cardiomyopathy including the probability of LV involvement. Modified from refs. [7] and [8] with permission.

Gene	Protein	Estimated % of cases	Inheritance	Expected LV involvement
*CDH2*	N-Cadherin-2	<2%	AD	–
*CTNNA3*	AlphaT-catenin	<2%	AD	–
*DES*	Desmin	<2%	AD	Frequent and often isolated
*DSC2*	Desmocollin-2	1%–8%	AD	If present, associated to RV involvement
*DSG2*	Desmoglein-2	3%–20%	AD/AR	Isolated or associated to RV involvement
*DSP*	Desmoplakin	3%–15%	AD/AR	Frequent either isolated or associated to RV involvement
*FLNC*	Filamin C	<2%	AD	Frequent and usually isolated (also a dilated biventricular cardiomyopathy phenotype can be present)
*JUP*	Plakoglobin	<1%	AD/AR	If present, associated to RV involvement
*LMNA*	Lamin A/C	<4%	AD	If present, associated to RV involvement
*PKP2*	Plakophilin-2	20%–46%	AD/AR	If present, associated to RV involvement
*PLN*	Phospholamban	<1%	AD	Frequent and often isolated
*SCN5A*	Sodium channel	2%	AD	If present, associated to RV involvement
*TGFβ3*	Transforming growth factor β3	<2%	AD	–
*TMEM43*	Transmembrane protein 43	<2%	AD	If present, associated to RV involvement
*TTN*	Titin	<10%	AD	–

AD: autosomal dominant; AR: autosomal recessive; LV: left ventricular; RV: right ventricular.

AC was first described in patients with a clear profound right ventricular (RV) structural changes with or without left ventricular (LV) involvement [6]. Since 2008 also left dominant AC (LDAC) forms have been identified [11–14] and certain genes have been more often been associated to them (Table 1).

From a histologic perspective, structural changes associated to IHD and AC may include fibrosis, fatty infiltration and myocardial loss, but certain of their characteristics and several additional features may help to distinguish both entities (Table 2).

**Table 2.** Pitfalls for the differential diagnosis of IHD and AC as the cause of death in postmortem of SCD victims. Put together by the authors, partially based upon refs. [5,6,12–14].

Cardiac condition	IHD	AC
LV fibrosis	Scars represent the sequelae of previous myocardial infarction. Replacement fibrosis at the scars usually involving the ventricular wall from subendocardium to the subepicardium (can be transmural). The scar coincides with an anatomical coronary distribution (sometimes several territories can be simultaneously affected).	Replacement with or without interstitial fibrosis involving the ventricular wall from subepicardium to the subendocardium (can be only midmyocardial or transmural). The scar typically affects the inferolateral wall, and sometimes extends to adjacent walls being occasionally completely circumferential.
LV fatty infiltration	Fatty infiltration is possible at the myocardial scars of old myocardial infarctions.	Fatty infiltration associates fibrosis and very often degenerated myocytes exhibit cytoplasmic lipid droplets.
Other features at the myocardium	Infrequently histological signs of acute or subacute myocardial infarction are identified when the ischemic insult precedes more than 4 h the development of the lethal ventricular arrhythmia causing the death (intramyocardial oedema, haemorrhage and neutrophilic infiltrates). RV is often preserved. Hypertensive heart disease with myocardial remodelling and intramyocardial small vessel disease are frequent.	Lymphocyte infiltrates (ranging from scarce to definite myocarditis) can be present.
Non-myocardial features	Atherosclerotic plaques are often identified, not always causing significant stenosis (>75%). Infrequently complicated plaques (eroded, ruptured, with or without thrombus) are identified. More frequent over 35 years and typically men with metabolic syndrome features (e.g. non-alcoholic fatty liver disease). Familial aggregation may be present.	In rare forms (autosomal recessive) woolly curly hair and keratosis palmoplantaris are extracardiac hallmarks of the disease. Wide variety of ages ranging from adolescents to middle aged, more men than women and often sports-triggered deaths. Familial aggregation is often present.

IHD: ischemic heart disease; AC: arrhythmogenic cardiomyopathy; SCD: sudden cardiac death; LV: left ventricular; RV: right ventricular.

Herein, we present a case of SCD due to LDAC which was initially misdiagnosed as IHD almost 1 decade ago, exactly when the LDAC forms were just starting to be recognized in the international literature. The review of the case upon the new knowledge recently accumulated reclassified the cause of the death to LDAC. The subsequent thorough family study not only diagnosed more individuals and allowed a tailored management, but also reassured others as healthy relatives. We highlight the importance of critical re-evaluation of previous postmortem reports since fortunately science keeps on moving and some previous diagnoses may not be accurate nowadays. When new diagnoses involve genetic conditions the beneficial effect of reclassifying those cases is crucial for at-risk relatives.

## Material and methods

Autopsies were performed at the Instituto de Medicina Legal y Ciencias Forenses de Valencia, Spain. Sudden death was defined as a natural death occurring within 1 h from the onset of symptoms in someone without a known severe condition which could explain such an abrupt outcome (in unwitnessed cases, the victim should have been checked to be in good health 24 h before death) [15, 16]. When a sudden death was assumed to be caused by a cardiac disease with a certain or a high probability, the death was classified as SCD. IHD-SCD was considered when at least one of the following criteria were present: an atheromatous stenosis >75% in at least one epicardial coronary artery, a complicated plaque (ruptured or eroded with or without thrombosis) and the presence of acute or healed myocardial infarction. AC postmortem diagnosis (at the reviewed autopsy) relied upon the finding of fibrofatty myocardial replacement with myocardial degeneration, as previously described. The macroscopic and histologic study included in the postmortem conformed the available guidelines at that moment [5, 6]. Hematoxylin-eosin and Masson trichrome stainings were examined in formalin-fixed paraffined-embedded myocardial blocks. The cardiological work-up in his first-degree relatives initially comprised an electrocardiogram (ECG) and an echocardiogram. Their extended cardiological evaluation 9 years later also included a cardiac magnetic resonance (CMR), an ECG-monitoring, and an exercise testing. A comprehensive genetic study with next generation sequencing (NGS) technology and copy variation number analyses (including 214 genes associated with inherited cardiac diseases) was performed in an affected individual with a typical LDAC phenotype, a proband’s distant relative. Once the desmoplakin rearrangement was identified in that sample, a predictive genetic testing was offered to the relatives in a cascade cardiogenetic fashion by means of a multiplex ligation-dependent probe amplification (MLPA) of the exons of the desmoplakin gene involved in the mutation. The pedigree was expanded accordingly and an extended cardiological work-up (ECG holter, exercise testing and CMR) was offered only to mutation carriers.

## Results

**Figure 1.**
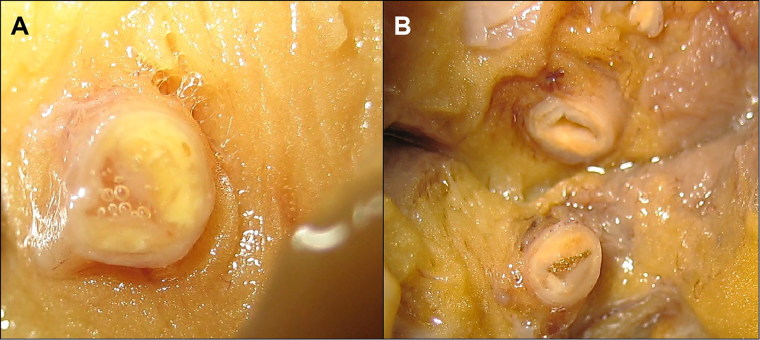
Coronary lesions at the postmortem. Atheromatous stenosis >75% of the left main (A) and circumflex (B) coronary arteries. Please note the atherosclerotic plaques with a lipid core.

**Figure 2.**
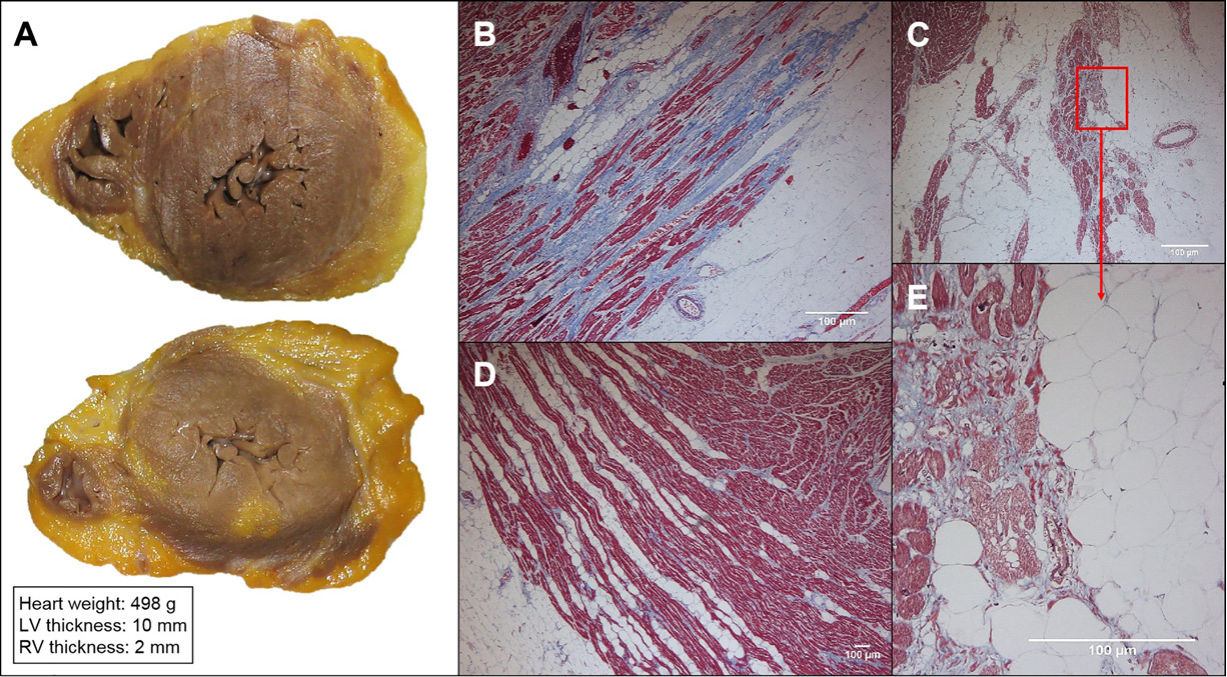
Macroscopic and histological study of proband’s heart. (A) Cross section of the heart near apex and the immediately above segment. Marked epicardial adipose infiltration with a thick subepicardial band at the left ventricle (LV). Microscopic images of the posterior (B) and lateral (C) wall of the LV that show subepicardial fibrofatty infiltration with different amounts of fibrosis. (D) Right ventricle (RV) showing mild lipomatosis in the external third myocardium, without myocardial degeneration or replacement fibrosis. (E) Detail of the myocyte degeneration.

**Figure 3.**
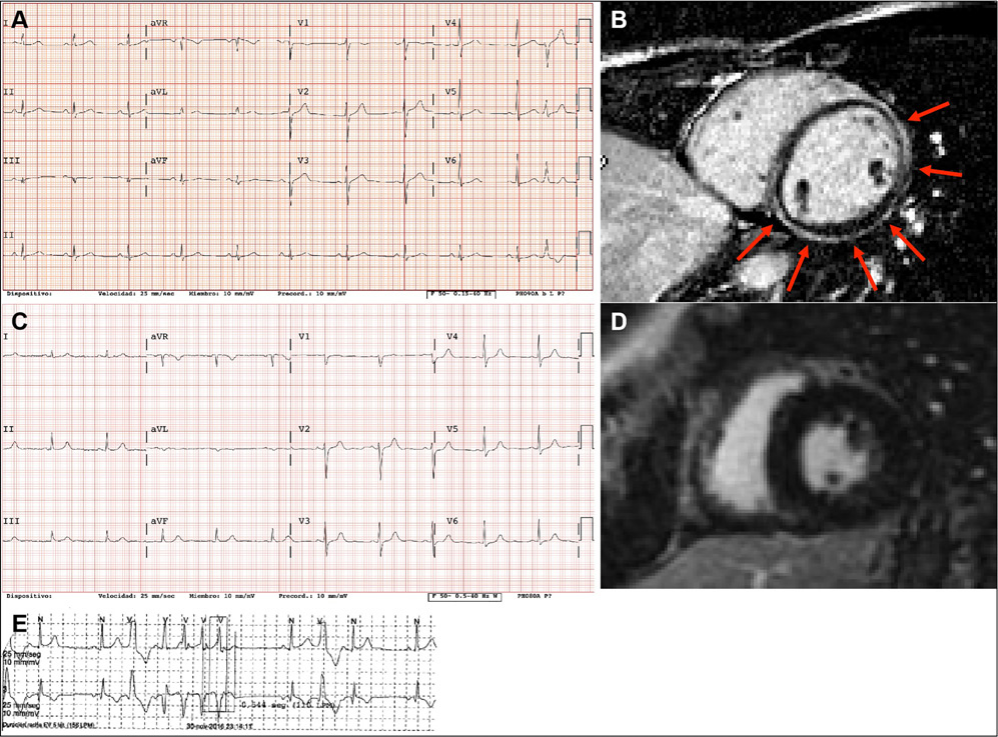
Images from the cardiological work-up in living mutation carriers. (A) Resting electrocardiogram (ECG) from the proband’s distant relative in whom the genetic study was performed, with a left dominant arrhythmogenic cardiomyopathy (LDAC) phenotype. (B) Subepicardial extensive late gadolinium enhancement in the same individual as in (A). (C) Resting ECG from the proband’s sister with palpitations. (D) Normal cardiac magnetic resonance imaging without late gadolinium enhancement in the same patient as in (C). (E) A run of non-sustained ventricular tachycardia recorded at the holter ECG in the same patient as in (C).

In 2008, an apparently healthy 37-year-old man died suddenly while playing basketball. The postmortem study was performed showing 75% stenosis of the left main and circumflex coronary arteries (Figure 1). Additionally, a significant amount of fatty tissue wrapped the whole heart and a band-like area of subepicardial fibrofatty infiltration with mild myocardial degeneration was identified in LV free wall corresponding to the anterior, lateral and inferior walls (Figure 2). At that point, these findings were assumed to reflect a past myocardial infarction in an adipositas cordis (as an anatomical variant of the normal heart), since also significant coronary stenoses had been clearly identified. His two sisters (the only living first-degree relatives) were offered a cardiological evaluation. They referred that their mother suffered from postpartum dilated cardiomyopathy (DCM) since she was 33 and died at age 54 due to several complications, while their father died at age 60 without any cardiomyopathy. Additionally, the maternal grandfather and several uncles suffered from heart diseases at advanced ages, and no heart transplantation or SCDs (except the proband’s) had occurred in the family. The two sisters were asymptomatic, exhibited no cardiovascular risk factors and their ECGs and echocardiograms remained within normal limits, thus they were discharged. Nine years thereafter, distant relatives from the maternal side of the proband’s family were clinically diagnosed with LDAC and confirmed with the identification of a radical mutation, namely a desmoplakin rearrangement. The proband’s two sisters were then re-evaluated and one of them, now symptomatic with palpitations, was found to have frequent ventricular ectopies with runs of non-sustained ventricular tachycardias without any structural involvement at the heart imaging (echocardiogram and CMR). The postmortem of the proband was then reviewed and reclassified as LDAC (based on the LV fibrofatty infiltration) with co-occurring significant and stable atherosclerotic coronary stenoses. RV AC involvement was ruled out since only a mild amount of fatty tissue was observed, without myocardial degeneration or replacement fibrosis (Figure 2(D)). Both the proband and his sister with ventricular arrhythmias were mutation carriers. The family study (Table 3, Figure 3) was completed stepwise yielding a total of 19 people studied, including one SCD due to LDAC and mutation carrier (the proband), three living patients diagnosed with LDAC and mutation carriers (two of them with a defibrillator), one mutation carrier with electrical but no structural involvement (one proband’s sister) and 14 healthy relatives not mutation carriers and with normal basic cardiological study (who were discharged).

**Table 3.** Family study. Only mutation carriers and obliged carriers are included.

	First-degree relatives mutation carriers (*n* = 1)	More distant relatives mutation carriers (*n* = 3)	Obliged mutation carriers (*n* = 4)
TFC 2010 or clinical data if TFC not available	Woman with borderline AC at 42 years old, normal CMR.	Man with definitive AC (LDAC) since 21 years old with severe LV LGE. Woman with definitive AC (LDAC) since 19 years old with severe LV LGE. Woman with possible AC at 63 years old with mild LV LGE.	Woman with DCM since her first pregnancy at 33 years old, dead at 54 years old. Man with cardiomyopathy, dead at 70 years old. Man with no history of heart disease, dead at 48 years old. Woman with no history of heart disease, SCD at 61 years old.
Clinical management	β-blockers, regular follow-up, cascade screening extended to her offspring.	β-blockers, heart failure optimal medical treatment, two ICDs, regular follow-up, genetic counselling for future pregnancies.	–

TFC 2010: Task Force Criteria released in 2010; AC: arrhythmogenic cardiomyopathy; CMR: cardiac magnetic resonance; LDAC: left ventricular dominant arrhythmogenic cardiomyopathy; LV: left ventricle; LGE: late gadolinium enhancement; DCM: dilated cardiomyopathy; ICD: implanted cardioverter-defibrillator; SCD: sudden cardiac death.

## Discussion

Johann Wolfgang von Goethe (1749–1832) once underlined the need to know in order to be able to recognize things (*We only see what we know*). Likewise in Medicine, only the awareness of a certain disease can open our eyes to recognize and diagnose it. Research moves forward the current knowledge of the illnesses, and their diagnostic criteria and tools change, so that sometimes diagnoses can change when new clues are depicted and diagnoses reconsidered. The high prevalence of IHD as the leading cause of SCD and the scarce (if any) published data concerning LDAC forms in 2008 prompted an inaccurate first diagnosis in the initial postmortem report of our proband. As a result of that, no genetic study was initially performed and the proband’s two sisters were discharged at a young age. Remarkably, these two decisions would not have been made in case of a solid diagnosis of AC in the proband [17, 18]. However, the family-based study performed in other relatives led us again to this family 9 years afterwards, once the LDAC forms had been widely recognized in the international literature and the diagnostic criteria for AC had been changed to include at least one feature (ventricular repolarization) of LV involvement [10]. Fortunately, we were able to complete the studies previously done so that the deceased proband was finally diagnosed of LDAC, one sister was identified as mutation carrier with need of indefinite cardiological reassessment and cascade cardiogenetic study was comprehensively expanded. Of note, we could prove that the proband’s mother had been an obligate mutation carrier and the referred history of postpartum cardiomyopathy presumably corresponded to an advanced biventricular AC form. The desmoplakin radical mutation showed a good co-seggregation with the AC phenotype in the whole family and tied in with the LV involvement without or with mild structural disease at the right ventricle (Tables 1 and 2). The finding of genetic rearrangements in AC-related genes is not common. Actually, 160 mutation negative AC patients underwent MLPA studies and 11 (6.9%) were found to harbour this type of genetic mutation often in the *PKP2* gene (nine individuals) and rarely in the *DSG2* or *DSC2* genes (one *DSG2* deletion and one *DSG2* and *DSC*2 simultaneous deletion) [19]. No genetic rearrangement in the *DSP* gene has been published so far.

## Conclusion

In summary, differential diagnoses are sometimes difficult in Medicine and may vary over time according with scientific endeavours. At a histological level, LV myocardial scars and fatty infiltration may be identified both in IHD and in AC though their distribution and additional features are helpful to distinguish both entities. At a family level, an IHD or an AC diagnosis has profound implications for defining further follow-up, genetic studies and counselling in at-risk relatives. Importantly, correct early diagnoses may help to prevent more SCD in the family.
